# The consequences of misinformation concern on media consumption

**DOI:** 10.37016/mr-2020-149

**Published:** 2024-06-25

**Authors:** Elizabeth A. Harris, Stephanie L. DeMora, Dolores Albarracín

**Affiliations:** (1) Annenberg Public Policy Center, University of Pennsylvania, USA

## Abstract

For the last decade, policymakers, journalists, and scientists have continued to alert us of the threat of misinformation for making sound decisions in the political, health, and environmental domains. In this study, we evaluate whether perceiving misinformation as a threat affects media use, particularly considering selection of media sources that are politically aligned. We show which groups are more likely to be concerned about misinformation and find experimental and correlational evidence of an impact of concern on greater use of politically aligned sources among Democrats. We also found no evidence that perceiving higher ability to detect misinformation decreases this association.

## Implications

*Misinformation concern*, or worry around misinformation prevalence or impact, varies across demographic groups and does not always correspond to the reality of misinformation prevalence. Although concern may encourage people to seek accuracy, which is valuable (e.g., Rathje et al., 2023; Pennycook et al., 2021), a high level of concern may not be on par with the reality of misinformation prevalence. For example, according to Jungherr and Rauchfleisch (2022), exposure to common journalistic coverage of misinformation, labeled as “alarmist discourse,” or reporting that is out of proportion with reality (see Carlson, 2020, for more on misinformation as a moral panic) creates a heightened perception of threat (Jungherr & Rauchfleisch, 2022). However, up to this point, knowledge about the potential negative effects of concern with misinformation is rather limited. One important finding is that exposure to misinformation and a media emphasis on misinformation prevalence decreases trust in the media (Ognyanova et al., 2020). However, actual information-seeking outcomes, such as implications for use of media sources, have not been ascertained.

Most Americans are aware of the existence of fake news, and this awareness better equips them to distinguish between true and fake news online. A survey from 2022 found that, across 19 countries, 70 percent of respondents reported that misinformation was one of the top threats faced by their country (Poushter et al., 2022). Adding to this high level of concern, more and more coverage of misinformation, especially when it is fear-inducing, leads people to feel even more threatened by misinformation (Jungherr & Rauchfleisch, 2022). In this article, we investigated whether this feeling of threat affects individuals’ usage of news sources. Unfortunately, when under threat, humans do not always seek information in an optimal way. In an ideal world, people who are worried about misinformation should use a broad range of sources to cross-reference any information they find. However, protection motivation theory (Floyd et al., 2000; Maddux & Rogers, 1983) suggests that people who perceive a threat strive to reduce it and avoidance is one way to accomplish this. For example, whenever possible, people avoid environments with high levels of contamination and evade encounters with menacing individuals on the street. Accordingly, people who feel that misinformation is a threat are likely motivated to avoid the misinformation. If people believe that the majority of misinformation comes from news sources that are misaligned with their political affiliation (e.g., a person who identifies as a Democrat may be politically aligned with news sources like MSNBC, Bill Maher, or Huffington Post and politically misaligned with sources such as Breitbart News, One America News, or The Drudge Report), greater misinformation concern should produce greater avoidance of politically misaligned sources.

Another common strategy to deal with an informational threat (Albarracín & Mitchell, 2004; Albarracín et al., 2012; Hart et al., 2009) is to seek information that agrees with one’s attitudes and beliefs. For example, people who are confronted with their own mortality seek information relevant to their worldview (Jonas et al., 2003). As another example, people who feel unable to defend their attitudes from external challenges are more likely to seek information that supports those attitudes (Albarracín & Mitchell, 2004; Albarracín et al., 2012). Therefore, we posited that greater concern with misinformation may result in exposure to media sources that are politically aligned with one’s views, also resulting in this selective exposure phenomenon. For example, a Democrat who is more concerned with misinformation would be more likely to use CNN, where they will find more liberal-leaning content, than would a Democrat who is less concerned with misinformation. In both cases, misinformation concern would lead to selective exposure, which has been shown to increase political polarization (Stroud, 2010).

In this research, we were interested in whether participants who feel more concerned with misinformation are more likely to seek news sources that align with their political affiliation. Across three studies, the last of which was longitudinal, participants reported their political party identification and how concerned they were with misinformation. Information about the items and data validating our measure of concern appear in the Methods section.

We also asked to what extent participants used a variety of media sources ranging from liberal- to conservative-leaning (Studies 2 and 3). We used Study 1 to assess who was more concerned about misinformation and found that Democrats, older people, and people with higher levels of education had higher levels of concern. In Studies 2 and 3, we found that Democrats used more liberal media sources when they were more (vs. less) concerned with misinformation. However, Republicans showed this association only in Study 2.

Due to the potential negative impact of concern with misinformation on politically aligned exposure to media, we were interested in examining if we could mitigate the negative impact of misinformation concern. Thus, Study 2 included a manipulation of the potential moderator of perceived ability to distinguish between true and fake news. Our hypothesis was that greater confidence in one’s ability to discern true from fake news may decrease the impact of misinformation concern on politically aligned source information seeking. However, we found no evidence that our manipulation of ability moderated the relation between concern and media use.

In sum, we hypothesized that people may *avoid* misaligned information and/or *seek* more aligned information when faced with the threat of misinformation. In our studies, we found that Democrats consistently used more aligned sources when they were more concerned about misinformation, providing support for the seeking hypothesis among Democrats. However, concern with misinformation only led to lesser use of misaligned sources in one study, again among Democrats. Largely, the results provide robust support for the seeking hypothesis among Democrats.

Given our findings, future work should explore how to reduce the impact of misinformation concern on information source seeking. For example, an accuracy nudge has been shown to reduce partisan biases in distinguishing between true and false news statements (Rathje et al., 2023). Therefore, this intervention may reduce the partisan political alignment in source choice as well. An implementation of this intervention may be to introduce threatening descriptions of misinformation prevalence or impact by reminding the audience of the importance of seeking accurate information.

## Findings

### Finding 1: Democrats, older, and more educated people tend to be more concerned about misinformation compared to their Republican, younger, and less educated counterparts. Democrats and Republicans are equally likely to consume mainstream (or center) media.

**Figure 1.**
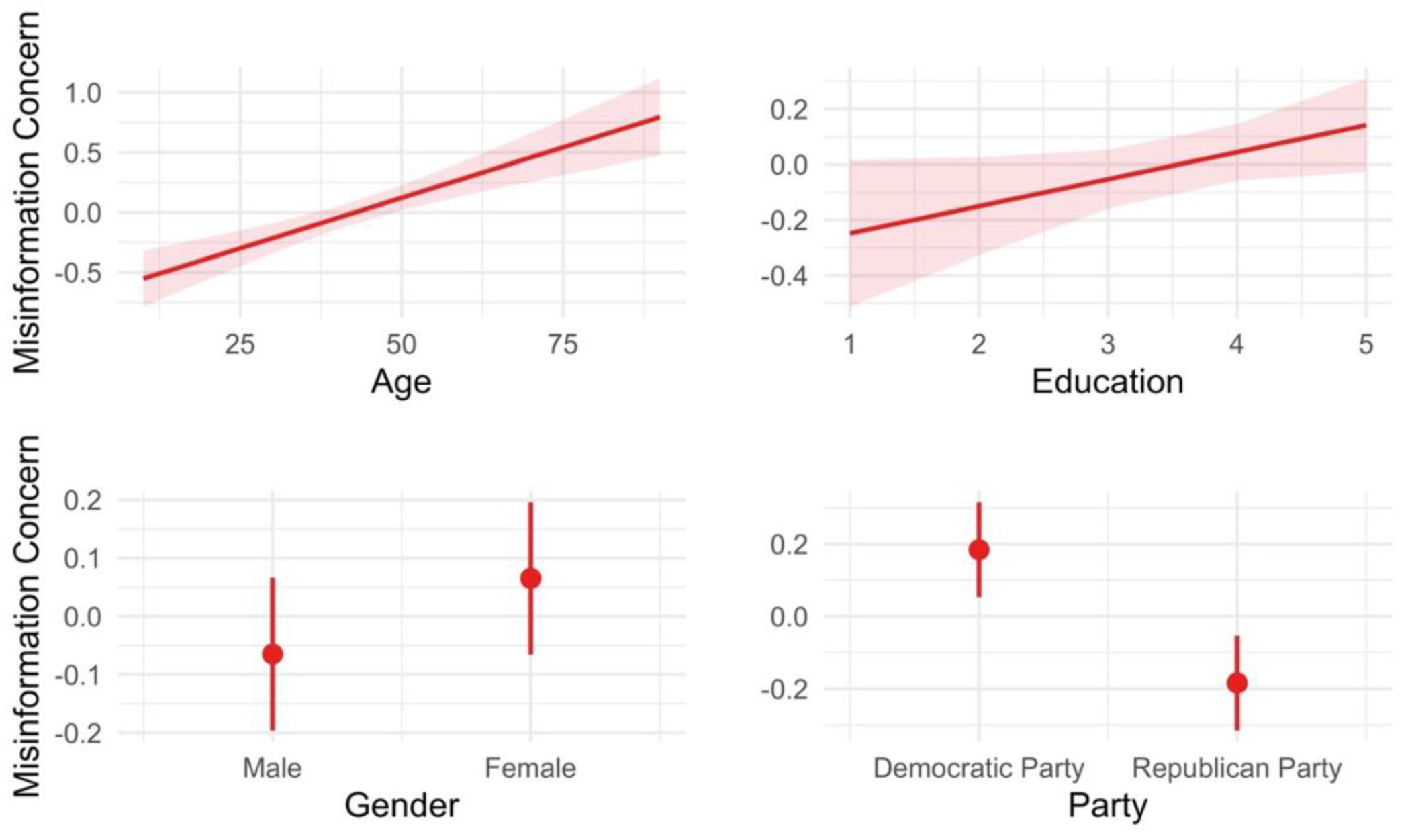
Study 1 estimated marginal means derived from an OLS linear regression model. The outcome is misinformation concern (y-axis).

In Study 1, we examined the basic descriptive differences in misinformation concern and media use among Democrats and Republicans. We also attempted to manipulate concern, but the manipulation had no effects (see Appendix Table A3), which led us to analyze only self-reported concern. Participants reported (a) how concerned they were with misinformation, (b) their basic demographic information (i.e., age, education, and gender), (c) their political partisanship, and (d) how frequently they planned on obtaining information from various media sources in the following week (e.g., “Sources such as MSNBC, Bill Maher, or Huffington Post” and “Sources such as Breitbart News, One America News, or The Drudge Report,” etc.). To assess the relation between misinformation concern and media use, we classified media sources according to their political lean. We classified “Sources such as Breitbart News, One America News, or The Drudge Report,” “Wall Street Journal (online or print),” and “Fox News” as politically aligned media for Republicans (i.e., conservative or conservative-leaning media). We classified “Sources such as MSNBC, Bill Maher, or Huffington Post,” “New York Times or Washington Post (online or print),” and “National Public Radio (NPR)” as politically aligned media sources for Democrats (i.e., liberal or liberal-leaning media). Finally, we classified “Sources such as ABC, CBS, or NBC News,” “Public TV station from your state or a nearby state,” and “Your local city or county newspaper (online or print)” as mainstream sources across both Democrats and Republicans. All of these classifications are based on study classifications by Albarracín et al. (2021) and AllSides Media Bias chart (see Appendix Table A1 for more information across studies). We then conducted an OLS linear regression of self-reported misinformation concern as a function of each demographic factor and the participants’ political party affiliation (Democrat or Republican). The results, which appear in Figure 1 and Appendix Table A3 column 1, showed the relation between misinformation concern and age (*B* = 0.02, 95% CI [0.01, 0.02], *p* < .001), and education (*B* = 0.10, 95% CI [0.00, 0.20], *p* < .05), sex (*B* = −0.13, 95% CI [−0.32, 0.06], *p* = .17), and partisanship (*B* = −0.37, 95% CI [−0.56, −0.18], *p* < .001). The results depicted in Figure 1 indicate that older people, more highly educated people, and Democrats are more concerned with misinformation than their younger, less educated, and Republican counterparts.

**Figure 2.**
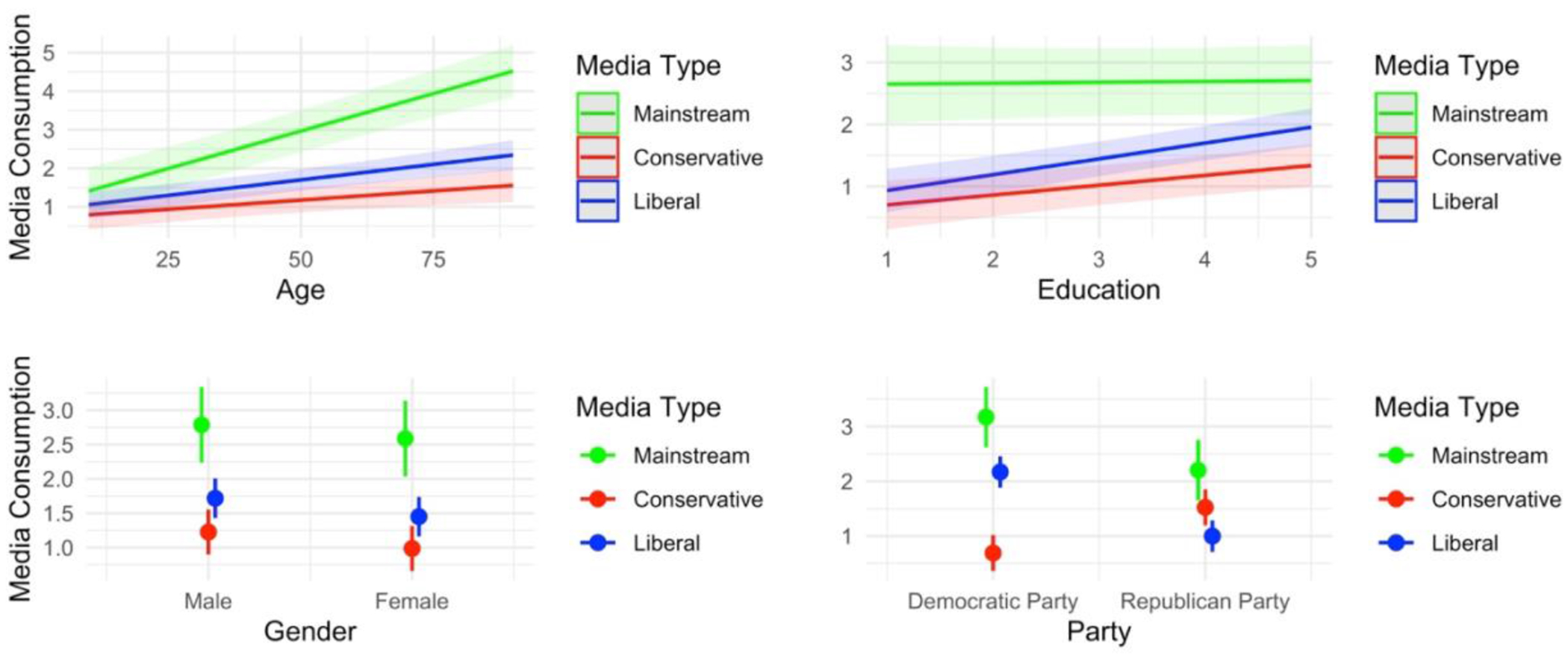
Study 1 estimated marginal means derived from an OLS linear regression model interacting each demographic with the media type. The outcome is misinformation concern (y-axis). Red lines and dot-whiskers are the mean values of conservative media consumption; blue lines and dot-whiskers are the estimated mean values of liberal media consumption; and green lines and dot-whiskers are the estimated mean values of mainstream (or center) media.

Next, we investigated the types of media respondents consume by age, education, gender, and partisanship. Specifically, we used a mixed-effects (hierarchical) regression to analyze media use as a function of the type of media interacted with respondents’ age, education, gender, and political affiliation. This model also included the individual and specific media sources as random effects. Figure 2 displays these results graphically, and the table of results is shown in Appendix Table A3 column 2. Most groups are quite similar in their consumption of different media types. Overall, respondents tend to consume more mainstream (i.e., center) media than either conservative or liberal media; both Democrats and Republicans use more mainstream media than media misaligned with their political affiliation; and men and women have very similar patterns of media use. However, two differences emerged. First, older participants consume more liberal media than younger participants. Second, whereas Democrats consume more mainstream media than politically aligned media, Republicans consume equal amounts of mainstream and politically aligned media.

### Finding 2: There is a positive correlation between misinformation concern and use of mainstream and politically aligned sources. There is no impact of perceived ability.

In Study 2, we again measured misinformation concern and political party as well as actual media use. We attempted to manipulate misinformation concern but our treatment did not result in any discernable effect. Therefore, we focused on the correlation between self-reported concern and media use as cross-sectional data rather than using the treatment as experimental. In addition, we manipulated participants’ perceived ability to distinguish between true and fake news (see Appendix Table A4). Using a mixed-effects regression model, we analyzed media use as a function of self-reported misinformation concern, the participants’ political affiliation (Democrat or Republican), the political lean of the media source (mainstream, liberal, or conservative), and the condition of manipulated perceived ability. We again included covariates for gender, age, and level of education, as well as random intercepts for participants and news sources. These analyses appear in Table 1. We found a significant 3-way interaction between self-reported misinformation concern, the participant’s political affiliation, and lean of the media source (liberal lean: *B* = 0.18, 95% CI [0.06, 0.29], *p* < .01; conservative lean: *B* = 0.31, 95% CI [0.18, 0.44], *p* < .001; see Appendix for more on model specification). These results hold in direction (*p* < .05) when we included a media quality variable calculated with measures by Lin et al. (2023; see Appendix Table A1 for more information on the quality scores). Additionally, the main effect and interactions, including manipulated perceived ability, were not significant (all p > .05).

Figure 3A presents the decomposition of the interaction for Democrats and Republicans in Study 2. Both Democrats and Republicans consume more mainstream media (Democrats: B = 0.41, 95% CI [0.32, 0.51], p < .001; Republicans: *B* = 0.11, 95% CI [0.02, 0.19], *p* < .05). Among Democrats, higher concern with misinformation was associated with more use of liberal-leaning sources (*B* = 0.22, 95% CI [0.14, 0.30], *p* < .001) but not conservative-leaning sources (*B* = 0.08, 95% CI [−0.02, 0.17], *p* = .10). Thus, this finding supported the idea that the Democrats were consuming politically aligned media and not consuming more politically misaligned media. However, there is not a negative relation between concern and politically misaligned media, meaning that concern does cause Democrats to actively avoid politically misaligned media.

Among Republicans, greater misinformation concern was correlated with more politically aligned media use (*B* = 0.08, 95% CI [−0.01, 0.17], *p* = .07), although the relation is marginally statistically significant. However, greater concern was also related to slightly more politically misaligned media use (*B* = 0.09, 95% CI [0.01, 0.16], *p* < .05). In summary, we again found support that higher concern is associated with Democrats and Republicans using more politically aligned media. However, in this study, greater concern with misinformation was associated with Republicans using more misaligned sources.

### Finding 3: There is support for a temporal effect of misinformation concern on media use.

In Study 3, we again asked participants to report how concerned they were about misinformation, their actual use of media sources, and their political party. But unlike Study 2, which was a single-time-point study, we obtained data at four time points over the course of nine months. This allowed us to have some confidence that our results were not being driven by certain kinds of people (e.g., high media consumers), and instead, analyze the relation between within-person change in concern and media consumption. To test the temporal nature of this relation, we conducted two multilevel models. The first was the same used in Study 1, but we included misinformation concern at times 1 and 2 and media use at times 3 and 4. We also included a fixed effect for time point. These analyses appear in Table 2. The second model included misinformation concern at times 3 and 4 as the outcome as a function of media use, media lean, and political party at times 1 and 2. This second model was conducted after reversing predictors and outcomes to rule out the possibility that media use might affect concern with misinformation. Table 2 and Figure 3B present the findings from the first model, which included media use as the outcome. As shown, this model revealed a significant 3-way interaction between misinformation concern, political party, and media lean (liberal media: *B* = −0.12, 95% CI [−0.18, −0.05], *p* < .001; conservative media: *B* = 0.21, 95% CI [0.12, 0.29], *p* < .001), supporting the conclusions from the previous two studies. In contrast, the second model had no significant effects, including a nonsignificant three-way interaction (liberal media: *B* = 0.002, 95% CI [−0.02, 0.01], *p* = .77; conservative media: *B* = −0.002, 95% CI [−0.02, 0.02], *p* = .85; see Appendix Table A5 column 2). The contrast between these two models thus supported our assumptions of a causal role of misinformation concern on actual media use.

The decomposition of the interaction from the model in Table 2 is shown in Figure 3B. As depicted, Democrats who were more concerned with misinformation used more politically aligned sources (*B* = 0.07, 95% CI [0.02, 0.11], *p* < .01). However, unlike Study 2, Democrats who were more concerned with misinformation used politically misaligned sources less (*B* = −0.24, 95% CI [−0.31, −0.16], *p* < .001), and concern was unrelated to mainstream media source use (*B* = −0.03, 95% CI [−0.06, 0.01], *p* = .18). In summary, we found temporal evidence that Democrats who were more concerned with misinformation at time 1 both sought more politically aligned media and avoided politically misaligned media at time 2. However, misinformation concern was not associated with media use among Republicans (mainstream: *B* = 0.02, 95% CI [−0.02, 0.05], *p* = .36; misaligned: *B* = −0.01, 95% CI [−0.06, 0.04], *p* = .70; aligned: *B* = 0.01, 95% CI [−0.03, 0.05], *p* = .65).

Finally, we include a series of robustness checks. First, we included a mean-centered measure of media quality (see Appendix Table A1 for descriptives) as both a main effect and in interaction with media source, but this addition had no impact on the direction and significance of our results. This new model dropped mainstream media sources because of its lack of variance in media quality. As in the main model, after media quality controls were introduced, Democrats who were more concerned with misinformation used politically aligned sources more (*B* = 0.07, 95% CI [0.01, 0.13], *p* < .05), and politically misaligned sources less (*B* = −0.20, 95% CI [−0.30, −0.11], *p* < .0001). Similarly, after media quality controls were introduced, we found temporal evidence that Democrats who were more concerned with misinformation at time 1 both sought more politically aligned media and avoided politically misaligned media at time 2. We further investigated the marginal effects of this interaction but did not find consistent results for quality of media sources across studies (see Appendix Tables A6, A7, and A8).

Second, we re-ran the analysis removing outliers (defined as responses on concern about misinformation and media consumption three standard deviations above or below the mean). Removing observations three standard deviations above or below the mean on both key variables resulted in a model that mirrored the results reported above. Democrats who were more concerned with misinformation used more politically aligned sources (*B* = 0.05, 95% CI [0.00, 0.10], *p* < .05). However, Democrats who were more concerned with misinformation used politically misaligned sources less (*B* = −0.27, 95% CI [−0.35, −0.19], *p* < .001), and concern was unrelated to mainstream media source use (*B* = −0.03, 95% CI [−0.07, 0.01], *p* = .17). As before, misinformation concern was not associated with mainstream media (*B* = −0.003, 95% CI [−0.05, 0.04], *p* = .87), aligned media use (*B* = 0.004, 95% CI [−0.05, 0.06], *p* = .87), or misaligned (*B* = −0.03, 95% CI [−0.09, 0.04], *p* = .46) media use among Republicans.

## Methods

### Study 1

We collected a large sample of participants using the platform Prolific, with the requirement that they be American and 18 years old or older. To obtain a balanced sample, we asked specifically for participants who identified as either Democrats or Republicans. In total, we collected 1,047 participants and removed 27 who identified as either independent or “other” at the time of our study. The remaining sample was composed of 512 Democrats and 508 Republicans, and a similar balance in gender (male = 507, female = 509, other = 4). The “other” responses were coded as NA due to the small number. Participants had a range of educational levels, including less than high school degree (*n* = 10), completed high school (*n* = 142), some college (*n* = 222), associate college degree (*n* = 88), bachelor’s degree (*n* = 393), master’s degree (*n* = 134), doctoral degree (*n* = 14), and professional degree (e.g., JD and MD; *n* = 17). The average age of our sample was 42.76 (*SD* = 14.36).

The design was correlational and included measures of our variables of interest: (a) self-reported misinformation concern, (b) use of mainstream, conservative, and liberal media sources, and (c) political affiliation (Democrat or Republican). First, we attempted to manipulate concern by having participants read either an article about the threat that misinformation poses or an unrelated threat. This experiment failed to produce statistically significant results. Therefore, participants were then asked to report their level of misinformation concern, followed by answering a series of items about media source use. They were then asked demographic questions including party affiliation. *Misinformation concern* was measured with reports of how concerned about fake news participants were, using a scale from 1 (not at all) to 7 (entirely). *Use of media* was measured with the item “How frequently do you think you will get information from each of these sources in the next week?” For all analyses in the main text, this variable was standardized (mean-centered). This was followed by a series of mainstream, liberal-leaning, and conservative-leaning media sources (e.g., “Sources such as MSNBC, Bill Maher, or Huffington Post” and “Sources such as Breitbart News, One America News, or The Drudge Report”; see Appendix Table A1 for the full list of sources and for more information about how we assigned “liberal-leaning,” “conservative-leaning,” and “mainstream” labels). For each group of media sources, participants rated their intended use of them from 0 days to 7 days. *Political party* was asked with the question, “With which political party do you most closely identify?” with the answer options being “Democrat,” “Republican,” “No party. I am independent,” or “Other Party.”

### Study 2

In Study 2, which was experimental, we collected a sample on the platform Prolific, requesting American participants 18 years old or older, half Democrat and half Republican. We collected 1,102 participants and removed 22 participants who, at the time of our study, identified as either independent or did not respond to the political affiliation question. The remaining sample was composed of 542 Democrats and 538 Republicans; 578 participants who identified as male, 496 who identified as female, and 6 “other” (again coded as NA). Participants’ educational levels included less than a high school degree (*n* = 10), completed high school (*n* = 133), associate or some college (*n* = 331), bachelor’s degree (*n* = 432), and more than college (*n* = 183). The average age of our sample was 40.31 (*SD* = 13.55).

The materials and procedures were very similar to those of Study 1 with a few small differences and the addition of the perceived misinformation ability manipulation. *Misinformation concern* was again measured by asking participants how concerned about fake news they were from 1 (not at all) to 7 (entirely). While we, again, attempted to manipulate concern, the treatments did not have an effect on concern across Democrats and Republicans; therefore, we used the self-reported measure of concern for these analyses. For all analyses in the main text, this variable was standardized (mean-centered). Political party was again measured with the question “With which political party do you most closely identify?” and using the same response options as in Study 1. The *use of media* measures used some different sources for generalizability (see Appendix Table A1) but was again measured by “In general, how frequently do you think you will get information from each of these sources in the next week?” Again, for each group of sources, participants rated their prospective use of them from 0 days to 7 days.

The main change in this study was the addition of the misinformation ability manipulation. For this, participants rated 10 headlines, half of which were actually true, as either true or false. Regardless of the real accuracy, a randomly assigned half of the participants were told “Congratulations, you got **9/10** correct! You did an excellent job distinguishing between true and fake news,” whereas the other half of participants were told “This should not be much of a concern, but you only got **2/10** correct. It is hard to distinguish between true and fake news.” At the end of the experiment, we introduced the manipulation check, “On a 7-point scale from Very Poor to Excellent, please tell us how good you are at distinguishing between true and fake news.” The manipulation was successful for both Democrats (*B* = 1.78, *SE* = 0.11, *t* = 16.85, *p* < .001) and Republicans (*B* = 1.85, *SE* = 0.11, *t* = 17.61, *p* < .001).

Other than the perceived ability manipulation, the procedure in Study 2 was very similar to Study 1. First, participants completed the misinformation ability manipulation, receiving either high or low ability feedback. They then answered the items about media source use, followed by a measure of their misinformation concern. We counterbalanced whether participants saw the media use or concern question first. Finally, we introduced the manipulation check and demographic questions, including party affiliation as a key predictor, as well as gender, age, and education as covariates in our models.

### Study 3

For Study 3, we collected a sample of participants (*N* = 2020; Americans 18 years old or older) through SSRS, a firm that runs a probability panel that reaches respondents via both phone and internet surveys. The data consisted of four time points (1: from July 7–23, 2022; 2: from September 7–21, 2022; 3: from December 7– 21, 2022; and 4: from March 8–23, 2023). We removed the 773 participants who identified as independent or “other” as well as those providing no response. The remaining sample (*N* = 1,247) was composed of 651 Democrats and 596 Republicans; 566 male, 670 female participants, and 8 “other” (coded as NA). Education was measured on a 4-point scale 1 (less than high school; *n* = 66), 2 (high school graduate; *n* = 395), 3 (some college; *n* = 340), and 4 (college degree +; *n* = 446). Age was similarly measured with brackets 1 (18-29; *n* = 223), 2 (30-49; *n* = 351), 3 (50-64; *n* = 326) and 4 (65+; *n* = 336). These are the demographics for the participants at time 1.

This study was very similar to Study 1, with the exception that it was longitudinal. However, our variables of interest were the same, namely misinformation concern, media use, and political party. *Misinformation concern* was measured by asking participants to report how concerned with fake news they were from 1 (not at all) to 5 (a lot). For all analyses in the main text, this variable was standardized (mean-centered). Use of media was measured with the item “How frequently, if at all, do you get information from [listed sources] each week?” with the listed sources being mainstream, liberal-leaning, and conservative-leaning media sources (e.g., “Sources such as MSNBC, Bill Maher, or Huffington Post” and “Sources such as Fox News, Breitbart News, One America News, or The Drudge Report”; see the Appendix Table A1 for the full list of sources). This is different from Studies 1 and 2, as they asked about media use *intentions* as opposed to Study 3 asking about *current* media use. These were rated on a scale from 0 days to 7 days. Political party was measured with the prior item with response categories: “Democrat,” “Republican,” “Independent,” and “Something else (not specified).” In terms of the order of the measures, participants first reported their misinformation concern, followed by media source use, followed by questions about gender, age, and education, which were introduced as covariates.

### Follow up study

All the analyses use a single measure of misinformation concern. However, in a follow-up study, we also asked five questions in addition to the measure included in each study. Specifically, in this validation study, we asked participants to “Please tell us to what degree you agree or disagree with the statement below” and listed the following statements: “I am concerned about fake news,” “I am concerned about the negative impacts of fake news,” “I am concerned about fake news harming our country,” “I am concerned about how quickly fake news spreads,” and “I feel concerned about the prevalence of fake news.” These measures showed a satisfactory level of internal consistency (Cronbach’s alpha = 0.74), which supported our interpretation that our single-item measure tapped a general sense of concern with misinformation.

## Figures and Tables

**Figure 3A.**
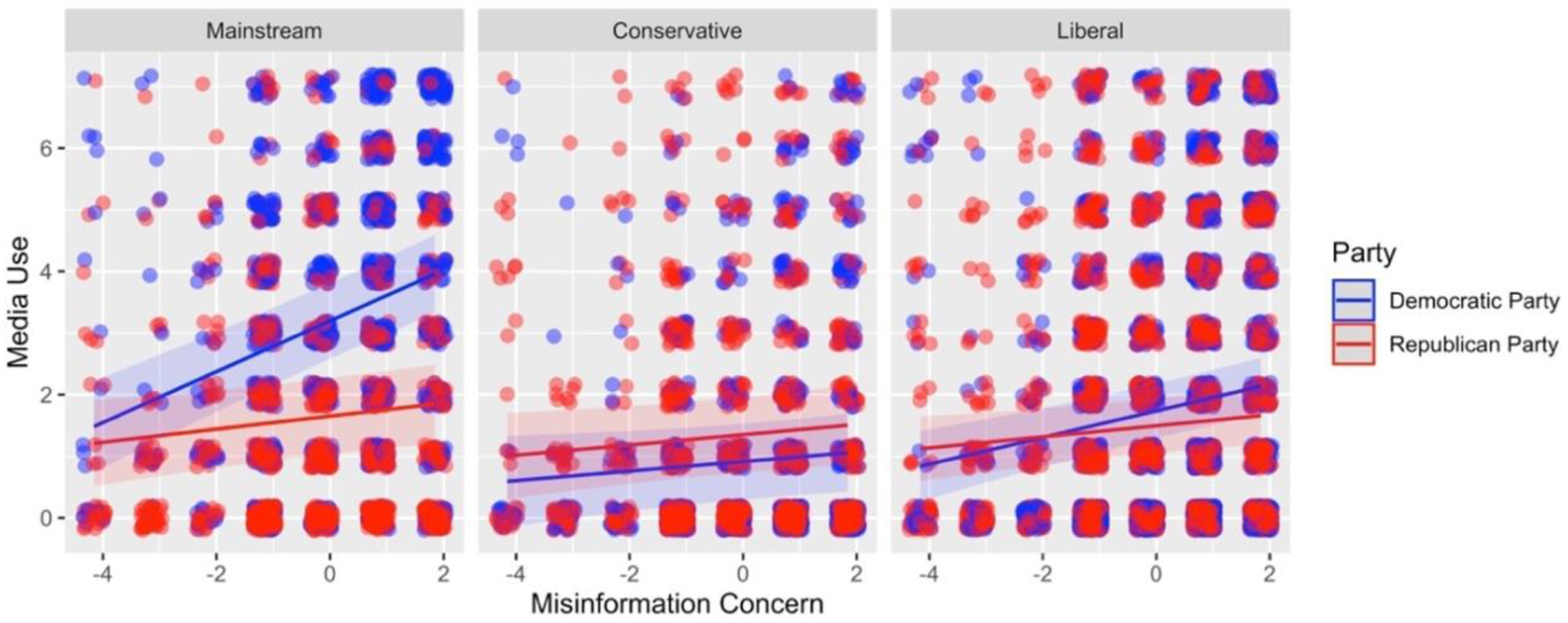
Three-way interaction Study 2. The interaction between misinformation concern (x-axis), party (line color), and media political lean (the three panels). The outcome is use of media from that category (y-axis).

**Figure 3B.**
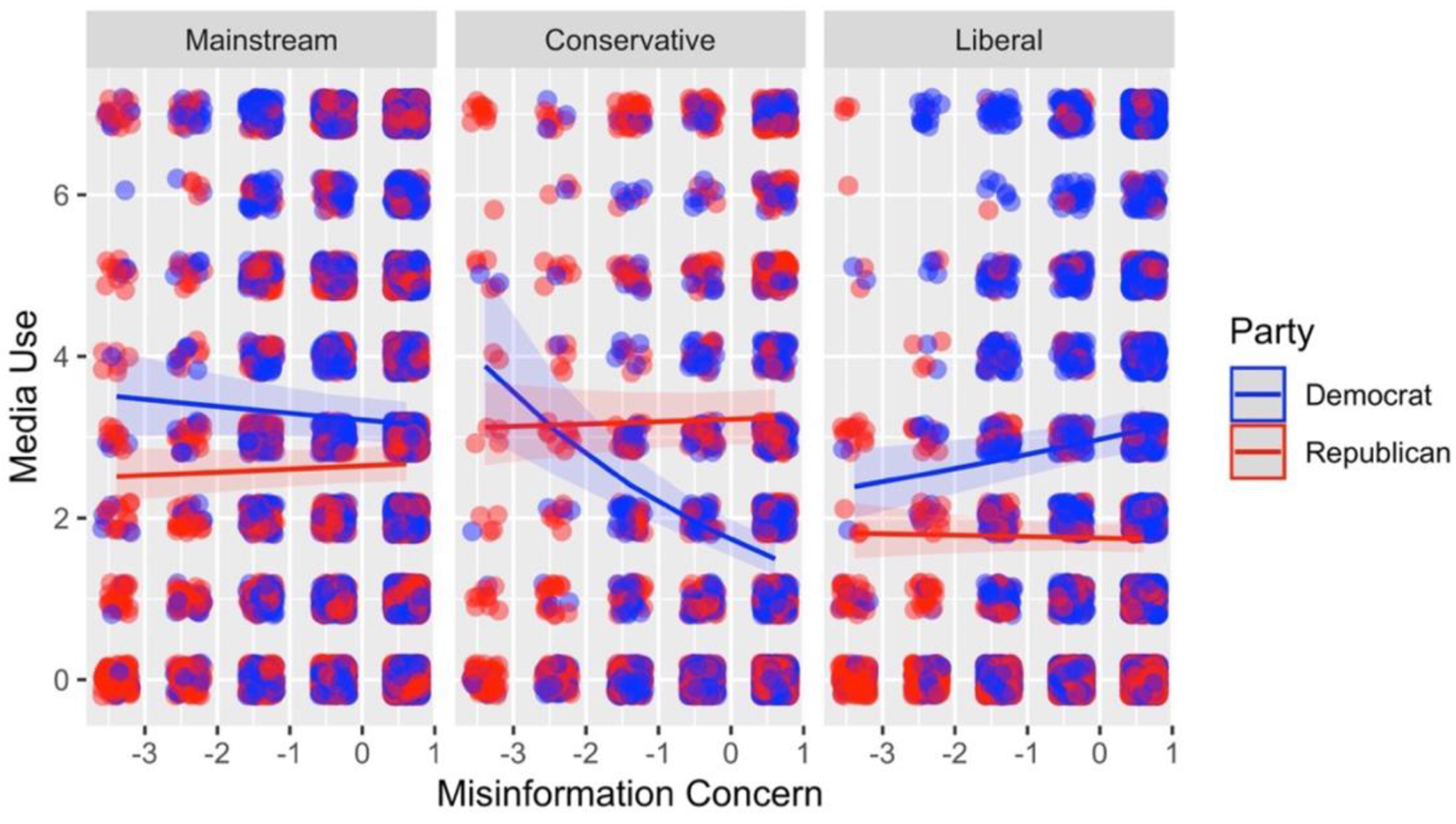
Three-way interaction Study 3 Model 1. The interaction between misinformation concern (x-axis), party (line color), and media political lean (the three panels). The outcome is use of media from that category (y-axis).

**Table 1.** Study 2 mixed (hierarchical) regression model.

	Mixed model on media consumption
(Intercept)	1.89 *** (0.36)
Concern	0.41 *** (0.05)
Republican	−1.55 *** (0.10)
Conservative Media Lean	−2.28 ** (0.44)
Liberal Media Lean	−1.46 * (0.38)
Efficacy	−0.06 (0.14)
Female	−0.29 *** (0.07)
Age	0.01 *** (0.00)
Education	0.22 *** (0.04)
Concern x Republican	−0.31 *** (0.07)
Concern: Conservative Media Lean	−0.34 *** (0.05)
Concern: Liberal Media Lean	−0.19 *** (0.04)
Republican: Conservative Media Lean	1.98 *** (0.10)
Republican: Liberal Media Lean	1.30 *** (0.09)
Concern: Efficacy	−0.04 (0.10)
Republican: Efficacy	−0.05 (0.19)
Conservative Media Lean: Efficacy	0.16 (0.14)
Liberal Media Lean: Efficacy	0.10 (0.12)
Concern: Republican: Conservative Media Lean	0.31 *** (0.07)
Concern: Republican: Liberal Media Lean	0.17 ** (0.06)
Concern: Republican: Efficacy	0.04 (0.13)
Concern: Conservative Media Lean: Efficacy	−0.00 (0.10)
Concern: Liberal Media Lean: Efficacy	0.07 (0.09)
Republican: Conservative Media Lean: Efficacy	−0.12 (0.20)
Republican: Liberal Media Lean: Efficacy	−0.06 (0.17)
Concern: Republican: Conservative Media Lean: Efficacy	−0.07 (0.14)
Concern: Republican: Liberal Media Lean: Efficacy	−0.17 (0.12)
N	8,568

Note:

*** p < 0.001;

** p < 0.01;

* p < 0.05.

Standard errors in parentheses.

**Table 2.** Study 3 mixed (hierarchical) zero-inflated negative binomial regression models.

	Media consumption
(Intercept)	0.64 *** (0.09)
Concern	−0.03 (0.02)
Republican	−0.19 *** (0.03)
Conservative Media Lean	−0.62 *** (0.06)
Liberal Media Lean	−0.08 (0.04)
Female	−0.01 (0.03)
Age	0.26 *** (0.02)
Education	−0.07 *** (0.02)
Time	0.01 (0.01)
Concern: Republican	0.04 (0.02)
Concern: Conservative Media Lean	−0.21 *** (0.04)
Concern: Liberal Media Lean	0.09 *** (0.02)
Republican: Conservative Media Lean	0.82 *** (0.05)
Republican: Liberal Media Lean	−0.33 *** (0.03)
Concern: Republican: Conservative Media Lean	0.21 *** (0.04)
Concern: Republican: Liberal Media Lean	−0.12 *** (0.03)
N	21,060

Note:

*** p < 0.001;

** p < 0.01;

* p < 0.05.

Standard errors in parentheses.

## Data Availability

All materials needed to replicate this study are available via the Harvard Dataverse: https://doi.org/10.7910/DVN/37A3QE.

## Research questions

- Is there a positive correlation between misinformation concern and use of politically aligned media sources?
- If there is a correlation, is it moderated by individuals’ perceived ability to distinguish between true and fake news?
- If there is a correlation between misinformation concern and use of politically aligned media sources, is misinformation concern prospectively linked to use of politically aligned sources?

## Essay summary

- To help answer our research questions we conducted three studies: the first helped us describe the population to be studied, the second was an experiment to answer research question two, and the third was a longitudinal study designed to answer research question three.
- Study 1 showed who is most concerned about misinformation (Democrats, older people, and people with higher levels of education). It also showed that all groups are more likely to consume mainstream media with some differences by age, education, and partisanship.
- Study 2 showed a positive influence of misinformation concern on the use of mainstream and politically aligned sources. We also found no evidence that perceived ability to distinguish between true and fake news moderated the impact of misinformation concern and use of politically aligned sources.
- Study 3 provided a longitudinal test of the association between misinformation concern and use of politically aligned sources, which was present only among Democrats.
- In conclusion, raising citizens’ concern with misinformation may increase polarization in media use among Democrats.

## Appendix: Supplementary information

**Table A1.** This table includes every news source from each study and whether they were rated as mainstream, liberal-leaning, or conservative-leaning. These ratings were made based on the ratings provided in Albarracín et al. (2021; see page 130; a combination of previous literature categorization, AllSides Media Bias, Fact Check, and Ad Fontes Media Index). We used AllSides media chart when a particular media source does not appear in Albarracín et al. (2021).

Sources	Lean	Studies the source appears in	Lin et al. (2023) quality score (ranging from 0 to 1)
Sources such as Breitbart News, One America News, or The Drudge Report	Conservative	Study 1	0.30, 0.41, 0.46 (avg. 0.39)
Sources such as MSNBC, Bill Maher, or Huffington Post	Liberal	Studies 1, 3	0.59, 0.72 (avg. 0.66)
Sources such as ABC, CBS, or NBC News	Mainstream	Studies 1, 2, 3	0.86, 0.88, 0.84 (avg. 0.86)
New York Times or Washington Post (online or print)	Liberal	Studies 1, 3	0.86, 0.82 (avg. 0.84)
Wall Street Journal (online or print)	Conservative	Studies 1, 3	0.80
CNN	Liberal	Studies 1, 3	0.66
Fox News	Conservative	Study 1	0.53
National Public Radio (NPR) and/or its local affiliates	Liberal	Studies 1, 2, 3	0.93
Sources such as Breitbart News, The Blaze, or The Drudge Report	Conservative	Study 2	0.30, 0.32, 0.46 (avg. 0.36)
Sources such as Daily Beast, Democracy Now, or Occupy Democrats	Liberal	Study 2	0.54, 0.57, 0.18 (avg. 0.43)
Sources such as MSNBC, CNN, or Huffington Post	Liberal	Study 2	0.59, 0.66, 0.72 (avg. 0.66)
New York Times (online or print)	Liberal	Study 2	0.86
New York Post (online or print)	Conservative	Study 2	0.49
Sources such as Fox News, Newsmax, or One America News	Conservative	Study 2	0.53, 0.29, 0.41 (avg. 0.41)
Sources such as Fox News, Breitbart News, One America News, or The Drudge Report	Conservative	Study 3	0.53, 0.30, 0.41, 0.46 (avg. 0.43)
Public TV station from your state or a nearby state	Mainstream	Studies 1, 2	NA
Your local city or county newspaper (online or print)	Mainstream	Studies 1, 2	NA

**Table A2.** This table includes the demographic breakdown of the samples of each study.

		Gender		Age		Education	

Study	Sample *N*	Male	Female	*M*	*SD*	*M*	*SD*
Study 1	1020	507	509	42.8	14.36	3.6	0.95
Study 2	1080	578	496	40.3	13.55	3.6	0.94
Study 3	1247	566	670	2.6	1.07	2.9	0.94

Notes: Study 3 is using the sample from time 1, and age is average age group (1-4). Education has been converted to a numerical variable (Study 1 and 3: 1-5, Study 2: 1-4), with higher numbers indicating a higher level of educational attainment.

**Table A3.** Study 1 OLS linear regression and hierarchical (mixed) models.

	OLS on concern	Mixed model on media consumption
	
(Intercept)	−0.95 *** (0.23)	1.56 *** (0.40)
Republican	−0.37 *** (0.10)	−0.97 (0.12)
Female	0.13 (0.10)	−0.20 (0.12)
Age	0.02 *** (0.00)	−0.04 *** (0.00)
Education	0.10 * (0.05)	0.01 (0.06)
Conservative Media Lean		−1.72 *** (0.43)
Liberal Media Lean		−0.84 (0.41)
Republican: Conservative Media Lean		1.80 *** (0.12)
Republican: Liberal Media Lean		−0.20 *** (0.12)
Conservative Media Lean: Female		−0.04 (0.12)
Liberal Media Lean: Female		−0.07 *** (0.12)
Conservative Media Lean: Age		−0.03 *** (0.00)
Liberal Media Lean: Age		−0.02 *** (0.00)
Conservative Media Lean: Education		0.14 * (0.06)
Liberal Media Lean: Education		0.24 *** (0.06)
	
N	1,016	1,016
R2	0.04	0.39
N (Participant)		1,016
N (News source)		10

Note:

*** p < 0.001;

** p < 0.01;

* p < 0.05.

Standard errors in parentheses.

**Table A4.** This table includes the results of the regression analysis of the Study 2 fake news perceived ability manipulation. The effect of the manipulation and political affiliation on perceived ability.

Predictor	*B*	*95% CI*	*SE*	*df*	*t*	*p*
(Intercept)	3.45	[3.30, 3.59]	0.07	1076	46.72	**< .001**
Democrat	0.18	[−0.02, 0.38]	0.10	1076	1.73	.084
Good feedback	1.85	[1.65, 2.06]	0.11	1076	17.61	**< .001**
Democrat * Good feedback	−0.08	[−0.37, 0.22]	0.15	1076	−0.52	.61

In Study 2, participants were asked to rate 10 headlines as true or false and were false feedback that either told them they did an excellent job or a poor job. The model predicted self-reported fake news ability with the interaction between participant political affiliation and which feedback they received. We found that the effect was significant for Democratic participants and for Republican participants.

To account for the overdispersion of zeros in the data, we specified a ZINB (zero-inflated negative binomial) regression model to analyze the results in our main text. However, no matter how limited the model, it failed to converge. Therefore, we use the mixed-effects model in Study 2 (see Table 1 in the main text), and used a ZINB model in our replication (Study 3; Table 2), which included more observations and successfully converged.

**Table A5.** This table includes the results of the second regression analysis of Study 3. The effects of concern, political affiliation, and lean of media sources on media use.

	Column 1	Column 2
	
(Intercept)	0.81 *** (0.11)	1.30 *** (0.04)
Concern	−0.02 (0.02)	
Republican	−0.41 *** (0.11)	−0.05 ** (0.02)
Time	−0.30 (0.16)	−0.00 (0.02)
Liberal Media Lean	−0.43 *** (0.10)	−0.01 (0.01)
Female	−0.01 (0.03)	0.01 (0.01)
Age	0.24 *** (0.02)	0.03 *** (0.01)
Education	−0.05 ** (0.02)	0.02 ** (0.01)
Time	−0.00 (0.01)	0.00 (0.01)
Concern: Republican	0.06 * (0.02)	
Concern: Conservative Media Lean	−0.07 (0.04)	
Concern: Liberal Media Lean	0.08 *** (0.02)	
Republican: Conservative Media Lean	0.55 ** (0.18)	0.00 (0.02)
Republican: Liberal Media Lean	0.17 (0.14)	0.01 (0.02)
Concern: Republican: Conservative Media Lean	0.03 (0.04)	
Concern: Republican: Liberal Media Lean	−0.11 *** (0.03)	
Media Use		−0.00 (0.00)
Media Use: Republican		0.00 (0.00)
Media Use: Conservative Media Lean		0.00 (0.01)
Media Use: Liberal Media Lean		0.00 (0.00)
Media Use: Republican: Conservative Media Lean		−0.00 (0.01)
Media Use: Republican: Liberal Media Lean		−0.00 (0.01)
	
N	20,954	20,954

Note:

*** p < 0.001;

** p < 0.01;

* p < 0.05.

Standard errors in parentheses.

In Study 3, we had three models. The results of the first model are reported in the main text, but the model reported in the main text includes more variables in the zero-inflated portion than the model in the table above. The model used in the main text also uses a standardized (mean-centered) primary independent variable, but the models in both of the columns here use the unstandardized versions. This is because our comparison model only converged with a minimal number of predictors in this second step, and so, for the sake of comparison, we limit both. Column 1 shows a model with the same time points and ordering of dependent and independent variables as in the main text. These models are limited to only the main predictor (a concern in column 1 and media use in column 2) and individual random effects and media-specific random effects across both models in the table above for proper comparison. For the model in column 2, we changed the time points and ordering of the variables to explore an alternate temporal order. This is to address the possibility that the relation between concern and media use is actually bi-directional (i.e., reciprocal). In this model, that did not appear to be the case. The model predicted misinformation concern at times 3 and 4 as a function of media use at times 1 and 2, media lean at times 1 and 2, and political party at times 1 and 2.

**Table A6.** This table includes the results of the primary analysis of Study 3 with the addition of media quality as part of the interaction term. The effects of concern, political affiliation, lean of media sources on media use, and media quality.

	Misinformation concern on media consumption
(Intercept)	0.12 (0.12)
Concern	−0.20 *** (0.05)
Republican	0.35 *** (0.07)
Liberal Media Lean	0.53 *** (0.04)
Media Quality	2.88 *** (0.18)
Time	0.03 (0.02)
Female	−0.07 (0.04)
Age	0.19 *** (0.02)
Education	−0.57 *** (0.07)
Concern: Republican	0.17 ** (0.06)
Concern: Liberal Media Lean	0.27 *** (0.04)
Republican: Liberal Media Lean	−0.90 *** (0.07)
Concern: Media Quality	1.10 *** (0.19)
Republican: Media Quality	−4.27 *** (0.23)
Liberal Media Lean: Media Quality	−2.78 *** (0.22)
Concern: Republican: Liberal Media Lean	−0.25 *** (0.06)
Concern: Republican: Media Quality	−1.28 *** (0.23)
Concern: Liberal Media Lean: Media Quality	−0.97 *** (0.24)
Republican: Liberal Media Lean: Media Quality	4.79 *** (0.35)
Concern: Republican: Liberal Media Lean: Media Quality	1.15 *** (0.34)
N	12,636

Note:

*** p < 0.001;

** p < 0.01;

* p < 0.05.

Breaking down this relation by high (one standard deviation above the mean) and low (one standard deviation below the mean) quality media, we found the negative association for Democrats’ conservative media consumption remains negative and statistically significant given low-quality conservative media (*B* = −0.380, *95% CI* [−0.467, −0.294], *p* < .0001), and while still negative, the relation is not statistically significant given high-quality conservative media (*B* = −0.025, *95% CI* [−0.153, 0.102], *p* = .697). Like in the main model, misinformation concern was not associated with media use among Republicans when media quality was average (misaligned: *B* = −0.003, *95% CI* [−0.070, 0.064], *p* = .926; aligned: *B* = −0.029, *95% CI* [−0.101, 0.043], *p* = .432). Similarly, the relation between misinformation concern and media consumption did not change in direction or statistical significance at any level of media quality. These results are shown in Table A7 below.

**Table A7.** Estimated marginal coefficients in Study 3 by media source and quality among Democrats and Republicans separately.

Party	Media Source	B	SE	Lower	Upper	Media Quality	p-value
Democrat	Conservative	−0.03	0.07	−0.15	0.10	1 SD Above	.70
Democrat	Conservative	−0.20	0.05	−0.30	−0.11	Mean	.00
Democrat	Conservative	−0.38	0.04	−0.47	−0.29	1 SD Below	.00
Democrat	Liberal	0.09	0.04	0.02	0.16	1 SD Above	.01
Democrat	Liberal	0.07	0.03	0.01	0.13	Mean	.03
Democrat	Liberal	0.05	0.05	−0.04	0.13	1 SD Below	.29
Republican	Conservative	−0.06	0.05	−0.16	0.05	1 SD Above	.29
Republican	Conservative	−0.03	0.04	−0.10	0.04	Mean	.43
Republican	Conservative	0.00	0.03	−0.05	0.05	1 SD Below	.99
Republican	Liberal	−0.00	0.04	−0.07	0.07	1 SD Above	.94
Republican	Liberal	−0.00	0.03	−0.07	0.06	Mean	.93
Republican	Liberal	−0.00	0.06	−0.12	0.11	1 SD Below	.95

Turning back to Study 2, the relation between concern about misinformation and media consumption for Democrats who consume aligned media is positive and statistically significant for average and high-quality media, the same as in Study 3. However, they also consume more high-average and high-quality misaligned media, which is very different from Study 3. The relation between concern about misinformation and media consumption for Republicans who consume aligned media is positive and statistically significant but only for low-quality media. Again, this is different from Study 3. These results are shown in Table 8 below. They also consume more high-average and high-quality misaligned media. Results for these quality of media sources are not consistent across the studies.

**Table A8.** Estimated marginal coefficients in Study 2 by media source and quality among Democrats and Republicans separately.

Party	Media Source	B	SE	Lower	Upper	Media Quality	p-value
Democrat	Conservative	0.15	0.07	0.01	0.28	1 SD Above	0.03
Democrat	Conservative	0.11	0.05	0.01	0.21	Mean	0.04
Democrat	Conservative	0.07	0.05	−0.03	0.16	1 SD Below	0.16
Democrat	Liberal	0.33	0.05	0.24	0.42	1 SD Above	0.00
Democrat	Liberal	0.17	0.04	0.08	0.25	Mean	0.00
Democrat	Liberal	0.01	0.06	−0.12	0.13	1 SD Below	0.94
Republican	Conservative	0.05	0.06	−0.07	0.17	1 SD Above	0.39
Republican	Conservative	0.07	0.05	−0.02	0.16	Mean	0.13
Republican	Conservative	0.09	0.04	0.00	0.17	1 SD Below	0.04
Republican	Liberal	0.09	0.04	0.01	0.17	1 SD Above	0.03
Republican	Liberal	0.09	0.04	0.01	0.16	Mean	0.02
Republican	Liberal	0.08	0.06	−0.03	0.20	1 SD Below	0.14
